# Structural and functional abnormalities in iron-depleted heart

**DOI:** 10.1007/s10741-018-9738-4

**Published:** 2018-10-03

**Authors:** Kamil A Kobak, Malwina Radwańska, Magdalena Dzięgała, Monika Kasztura, Krystian Josiak, Waldemar Banasiak, Piotr Ponikowski, Ewa A Jankowska

**Affiliations:** 1 Laboratory for the Applied Research on Cardiovascular System, Department of Heart Diseases, Wroclaw Medical University, Wrocław, Poland; 2 Department of Heart Diseases, Wroclaw Medical University, Wroclaw, Poland; 3 Centre for Heart Diseases, Military Hospital, Wroclaw, Poland

**Keywords:** Iron deficiency, Myocardial iron deficiency, Heart failure, Iron supplementation

## Abstract

Iron deficiency (ID) is a common and ominous comorbidity in heart failure (HF) and predicts worse outcomes, independently of the presence of anaemia. Accumulated data from animal models of systemic ID suggest that ID is associated with several functional and structural abnormalities of the heart. However, the exact role of myocardial iron deficiency irrespective of systemic ID and/or anaemia has been elusive. Recently, several transgenic models of cardiac-specific ID have been developed to investigate the influence of ID on cardiac tissue. In this review, we discuss structural and functional cardiac consequences of ID in these models and summarize data from clinical studies. Moreover, the beneficial effects of intravenous iron supplementation are specified.

## Introduction

Iron is one of the most important microelements in the body as it is involved in diverse metabolic processes, including transport of oxygen, synthesis of deoxyribonucleic acid (DNA) and oxidative energy production within the electron transport chain (ETC) [1]. With regard to its redox-based properties, iron is an integral part of Fe-S metalloproteins—key players in cellular energetics, which can be found in the nucleus, cytosol and mitochondria [2]. The optimal iron supply is critical particularly for the proper functioning of high-energy demand cells, specifically cardiomyocytes and skeletal myocytes [3, 4]. It is well established that either iron overload or ID unfavourably affect human condition and in particular, the role of disordered iron homeostasis in heart failure (HF) has been elucidated in several papers [5–9]. This review will focus on structural and functional abnormalities occurring in the heart as consequences of systemic or cardiac-specific ID with the strong emphasis on molecular evidence.

## Systemic iron deficiency and its consequences for heart

ID is one of the most common nutrition deficiencies worldwide [10] and untreated may lead to ID related anaemia (IDA). The effects of systemic ID on cardiac function have been evaluated in several animal studies. Acute and chronic models of systemic ID have been developed in animals by bleeding or by feeding a low-iron diet, respectively [11–17]. The results have shown that ID with concomitant anaemia resulted in cardiomegaly, left ventricular (LV) hypertrophy, cardiac fibrosis and symptomatic HF [9, 14, 18, 19], leading in severe cases to hypervolemia and pulmonary congestion [14, 20, 21].

In animal models, dietary iron restriction causes a significant decrease of total heart iron [22] and numerous structural and ultrastructural aberrations in heart, specifically cardiomyocyte enlargement, degeneration of myofilaments, irregular sarcomere organization and mitochondrial swelling, with the latter two being accompanied by elevated expression of endothelial and inducible nitric oxygen synthase (eNOS and iNOS, respectively) and several stress-related protein molecules [16, 23]. Interestingly, the oxidative/nitrosative stress which is associated with elevated generation of reactive oxygen species (ROS) and/or reactive nitrogen species (RNS) is increasingly being recognized as one of the pathophysiological mechanisms of LV remodelling responsible for HF progression [24]. Additionally, Dong et al. have shown that ID in rats upregulated an expression of apoptosis-promoting protein caveolin-1 in myocardium and also triggered mitochondrial-to-cytosolic translocation of cytochrome c, which constitutes both an indicator of mitochondrial damage and the major pre-apoptotic mitochondrial phenomenon [23, 25, 26]. Further, in myocardium, ID also contributes to the impaired mitochondrial oxidative energy production within ETC, as the activities of the respiratory chain enzymes such as Complexes I-II and IV, succinic cytochrome c reductase (SCCR) and NADH ferricyanide oxidoreductase are reduced as well as mitochondrial oxygen uptake is lower [27]. Importantly, there is a study reporting that intravenous (i.v.) iron sucrose treatment of dietary iron-restricted mice reversed LV remodelling and prevented cardiac fibrosis, despite the fact that it did not fully correct IDA. Furthermore, i.v. iron reduced nitrosative and oxidative stress and attenuated inflammation in the heart [13].

## Cardiac-specific iron deficiency

**Fig. 1:**
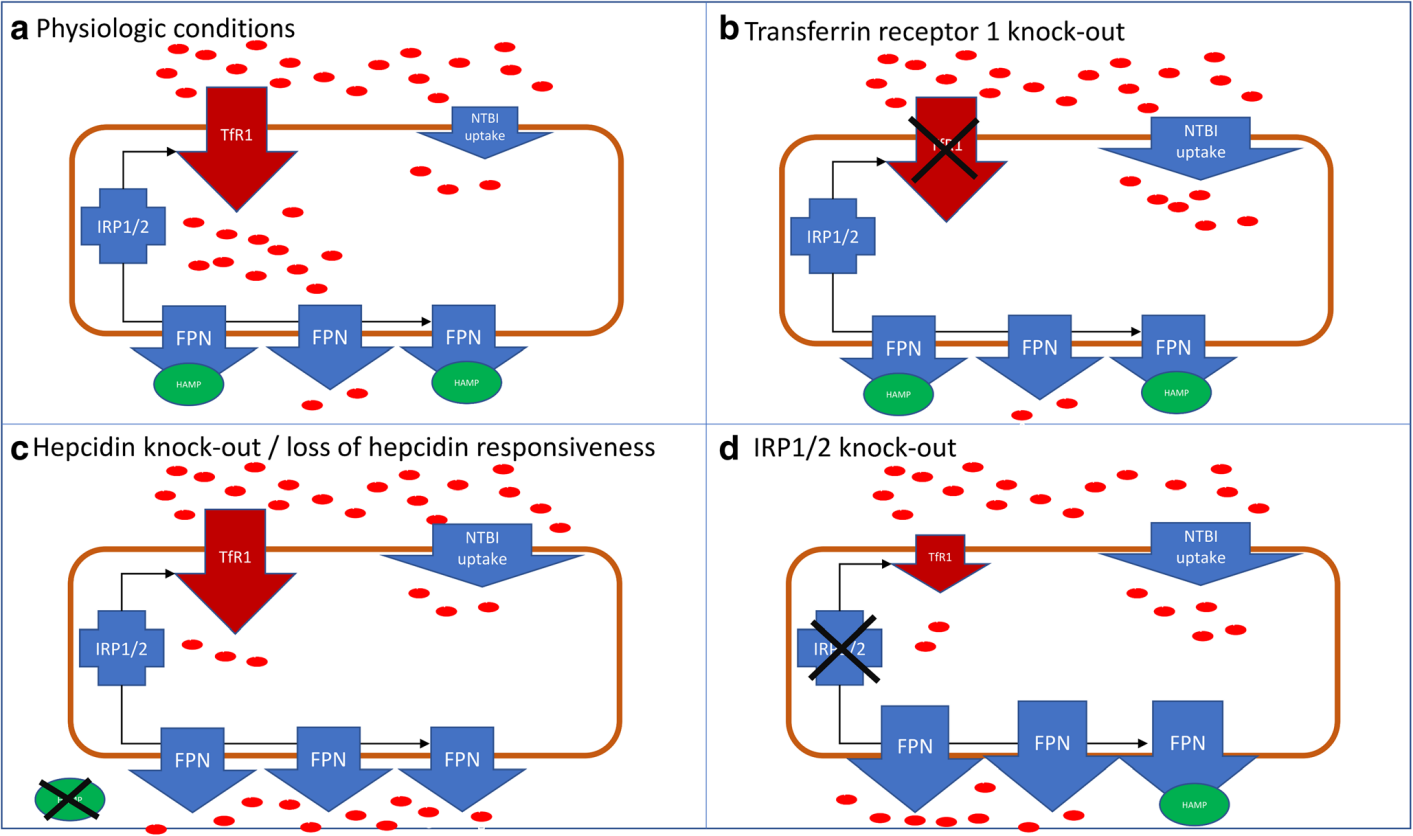
Molecular elements of intracellular iron metabolism in cardiomyocytes in different transgenic models of cardiac specific ID. **a** Physiologic conditions, **b** TfR1 knock-out, **c** Hepcidin knock out/loss of hepcidin responsiveness, **d** IRP 1/2 knock-out.  iron; TfR1–transferrin receptor 1; IRP1/2–iron regulatory proteins 1 and 2; FPN–ferroportin; HAMP–hepcidin; NTBI–non-transferrin-bound iron

Although the dietary iron restriction studies provided evidence on abnormalities in the heart muscle induced by systemic ID, it was extremely difficult to define the actual functional implications of myocardial iron deficiency (MID) independent of systemic ID and/or anaemia. Currently, there is a considerable interest in developing animal models of cardiac-specific ID in the absence of anaemia in order to circumvent the ambiguity in interpretation. In this paragraph, we discuss different approaches applied to induce cardiac-specific ID and present recent findings in this field (Fig. 1).

### Cardiomyocyte-specific *Tfr1* knock-out

Ubiquitously expressed transferrin receptor 1 (Tfr1) is a membrane glycoprotein that serves as a gatekeeper in regulating cellular uptake of iron from transferrin [28, 29]. Total Tfr1 knockout embryos were abnormal and died before embryonic day 12.5 with oedema and diffuse necrosis in all tissues consistent with anaemia and hypoxia. However, they presented no anatomical defects in the embryonic heart, suggesting that cardiac structures did not require Tfr1 to development [30].

To determine the importance of TfR1 in the heart, Xu et al. disrupted Tfr1 using a heart-specific promoter [31]. Tissue non-haemic and total iron concentration in Tfr1^hrt/hrt^ hearts was decreased at birth and did not change substantially during the post-natal period. Mice died by post-natal day 11 with cardiac hypertrophy and cardiomegaly. Tfr1^hrt/hrt^ hearts showed left ventricular dilatation, decreased fractional shortening, enlarged cardiomyocytes and significantly increased cardiac hypertrophy biomarkers, indicating that cardiomyocyte-specific TfR1 knockout leads to the phenotype of dilated cardiomyopathy. Fe-S cluster biogenesis, estimated by decreased expression of Dpyd and Ppat proteins [32], was compromised due to ID or mitochondrial dysfunction. Mitochondria from Tfr1^hrt/hrt^ hearts were also severely disrupted and enlarged, and expression and activity of ETC mitochondrial complexes (I-IV, where iron is necessary [33]) were significantly decreased. Notably, non-iron-containing Complex V expression and activity remained unchanged. Moreover, mitochondria-encoded mRNA levels were decreased, indicating fewer mitochondria or mitochondria unable to normal gene expression. mRNA expression profile also showed downregulation of genes associated with peroxisome proliferator-activated receptor signalling (PRAR) and PGC1-a (stimulator of mitochondrial biogenesis), myogenesis and insulin signalling as well as upregulation of hypoxia-inducible targets, Myc targets and glycolytic enzymes. Tfr1^hrt/hrt^ hearts also showed metabolic changes, increased apoptosis in addition to impaired mitophagy.

The survival of Tfr1^hrt/hrt^ mice has been prolonged by cardiac iron repletion via intravenous (i.v.) treatment with iron dextran, but mice still died at 4–5 weeks with severe cardiomegaly. However, iron-supplemented Tfr1^hrt/hrt^ mice assimilated and used given iron which was reflected by the fact that at day 10, Fe-S proteins (Dpyd and Ppat) as well as ETC proteins were at the similar level compared to WT mice. Moreover, heart-to-body weight ratios in iron-treated Tfr1^hrt/hrt^ mice after 10 days were similar to WT littermates. Notably, the administration of a second dose of dextran delayed the pathological changes and consequent death up to 13 weeks.

### Cardiomyocyte-specific HAMP knockout and FPN resistant to HAMP knock-in

Systemic iron homeostasis is regulated by the hepcidin-ferroportin interactions at the sites of iron release into the circulation. At this moment, ferroportin (FPN), also termed as Slc40a1, MTP1 or Ireg1, is the only known mammalian efflux channel for iron, which is localized on the surface of hepatocytes, enterocytes and macrophages—the cells responsible for iron storage, absorption and recycling, respectively [34]. The rate of FPN-mediated iron release depends on the plasma level of iron regulatory hormone hepcidin (HAMP, hepcidin antimicrobial peptide), which is known to inhibit FPN as a part of a homeostatic negative feedback loop [35]. Generated predominantly in liver, hepcidin binds FPN and triggers its endocytosis and subsequent degradation, which results in limited iron efflux into circulation and its lower availability to peripheral tissues [36]. Surprisingly, hepcidin is also expressed in other tissues, such as heart [37], kidney [38], brain [39] and placenta [40]. The exact role of this locally produced hepcidin is still unclear; however, there is some evidence that it may be associated with tissue-specific iron regulation [4, 41].

Lakhal-Littleton et al. have recently discovered expression of FPN in cardiomyocytes, where it was necessary for maintenance of iron homeostasis. Furthermore, the cardiomyocyte-specific deletion of FPN resulted in fatal cardiac iron overload in mice [42]. Given that, they further speculated that cardiac FPN may be under the control of cardiac HAMP and that their interaction may be essential for regulation of local iron homeostasis and proper cardiac function. Having first demonstrated that HAMP is expressed in cardiomyocytes, two novel mouse models were next generated: the one carrying a cardiomyocyte-specific deletion of the *Hamp* gene (*Hamp*^*fl/fl*^*;Myh6.Cre+* genotype) [43] and the other with a cardiomyocyte-specific knock-in of *Slc40a1 C326Y* gene (*Slc40a1 C326Y*^*fl/fl*^*;Myh6.Cre+* genotype), which encoded FPN completely resistant to hepcidin inhibition but with preserved iron export function [44, 45].

The results showed that in *Hamp*^*fl/fl*^*;Myh6.Cre+* mice, the cardiac Hamp mRNA and HAMP protein were almost undetectable, while the liver levels of mRNA and HAMP protein as well as liver iron stores and circulating markers of iron homeostasis remained intact. This is the first evidence that cardiomyocytes are the essential site of hepcidin expression in the heart and that systemic iron homeostasis is not influenced by cardiac hepcidin depletion. Moreover, the hearts of both *Hamp*^*fl/fl*^*;Myh6.Cre+* and *Slc40a1 C326Y*^*fl/fl*^*;Myh6.Cre+* mice developed deadly LV dysfunction characterized by reduced LVEF as well as enlargement of cardiomyocytes in addition to significantly greater mortality when compared to controls. The loss of cardiac HAMP stimulated upregulation of cardiomyocyte FPN in the hearts of both *Hamp*^*fl/fl*^*;Myh6.Cre+* and *Slc40a1 C326Y*^*fl/fl*^*;Myh6.Cre+* mice which in turn resulted in increased iron efflux that finally lead to cardiomyocyte ID. Additional studies revealed the reduced activities of iron-containing metabolic enzymes, signs of mitochondrial failure, significantly greater apoptosis and increased glycolysis in *Hamp*^*fl/fl*^*;Myh6.Cre+* hearts.

However, the most striking discovery was that all morphological abnormalities and metabolic and contractile dysfunctions were entirely suppressed by intravenous iron treatment [43].

### Cardiomyocyte-specific Irp1 and Irp2 knock-out

Maintenance of intracellular iron content is secured by two cytoplasmic iron-regulatory proteins 1 and 2 (IRP1 and IRP2, also known as ACO1 and IREB2) [46]. Cellular ID stimulates binding of IRPs to cis-regulatory iron-responsive elements (IREs) in the 5′ or 3′ untranslated regions of target mRNAs encoding proteins responsible for iron import (transferrin receptor, TfR1; divalent metal transporter 1, DMT1), sequestration (ferritin H- and L-chains; FTH1, FTL, respectively) and export (ferroportin) [47]. IRPs interact with 5′ IRES in ferroportin and ferritin mRNAs to inhibit their translation and with 3′ IREs in TfR1 to increase its stability and to protect from degradation [48]. In that manner, IRPs promote iron efflux and increase its availability for production of haem- and iron-sulphur proteins in cytosol or mitochondria [47]. Conversely, when intracellular iron is in excess, IRP1 loses ability to bind IREs, and instead binds an iron-sulphur cluster to start functioning as an aconitase enzyme, while IRP2 is targeted for proteasomal degradation [49]. IRP activity is significantly reduced in LV tissue in patients with advanced HF and LV tissue ID [50].

Functional implications of ID in the heart independently of systemic ID and anaemia were recently investigated by Haddad et al. [50]. In the study, cardiomyocyte-specific deletion of Irp1 and Irp2 (Cre-Irp1/2^f/f^) in mice resulted in significant reduction of iron concentration in LV and also in isolated cardiomyocytes with no evident change in the phenotype under the baseline conditions. Interestingly, under acute dobutamine hemodynamic stress, Cre-Irp1/2^f/f^ mice exhibited functional abnormalities, in particular inability to increase LV systolic function. After induction of myocardial infarction (MI), Irp-targeted mice presented more pronounced LV hypertrophy, greater increase in cardiomyocyte size and higher expression of embryonic marker genes. Additionally, LV dilatation and systolic dysfunction were more evident than in control mice. Furthermore, Cre-Irp1/2^f/f^ mice exhibited symptoms of ongoing HF failure, such as pulmonary congestion, accumulation of serous fluid in the chest cavity and increased mortality. Considering cardiomyocyte mitochondria, there were no significant differences between IRP-targeted mice and controls. However, the activity of ETC Complex I was significantly decreased, while the activity of complex IV was not affected. The oxygen consumption rate (OCR) in cardiomyocytes was not remarkably different in baseline conditions, showing that ATP production was comparable in both Cre-Irp1/2^f/f^ and control mice. However, upon maximal respiration, OCR increase in IRP1/2-deficient mice was strongly weakened when compared to the control mice. At baseline conditions, glycolytic activity of IRP-targeted mice was not affected; however, in maximal respiration, it increased to a smaller extent compared to control. In vivo cardiac energy metabolism study by ^31^P-magnetic resonance spectroscopy did not show any differences in PCr/ATP ratio in LV between Cre-Irp1/2^f/f^ and control. Nevertheless, after dobutamine stress, PCr/ATP ratio fell down significantly only in Cre-Irp1/2^f/f^, indicating limitation of high-energy phosphate deposits in IRP cardiomyocyte-knockout.

Noteworthy, cardiomyocyte single IRP1- or single IRP2-targeted mice did not develop MID and did not exhibit any of aforementioned symptoms.

Intravenous ferric carboxymaltose (FCM) injection restored both iron concentration in IRP-targeted left ventricle and OCR in maximal respiration in Cre-Irp1/2^f/f^ cardiomyocytes as well as restituted systolic function in dobutamine-stimulated Cre-Irp1/2^f/f^ mice. Moreover, i.v. iron treatment additionally prevented from increased hypertrophy after MI. FCM treatment in post-infarct IRP-targeted mice also improved LV systolic function and attenuated LV dilatation.

## Iron homeostasis and deficiency in the failing human heart

In patients with HF, the role of ID in pathophysiology is highly pronounced and deeply investigated. Accordingly to the broad definition of ID (serum ferritin < 100 μg/l or serum ferritin 100–300 μg/l in combination with TSAT < 20%), up to 50% of chronic HF patients suffer from ID (57% of anaemic and 32% of non-anaemic) [7, 51]. ID is more prevalent in women sex and in those with advanced NYHA class, higher serum levels of N-terminal pro-type B natriuretic peptide (NT-pro-BNP) and higher serum concentrations of C-reactive protein (CRP) [51]. ID is considered as a predictor of unfavourable outcome and is associated with impaired exercise capacity, independently of the presence of anaemia [6–8, 51]. In acute HF, where ID is also highly prevalent [52], ID was found as a predictor of increased mortality in both anaemic and non-anaemic patients [5, 53].

Recently, Melenovsky et al. performed a direct tissue analysis of myocardial iron content and mitochondrial function in HF [54]. As it has been showed before [18, 55], iron content in LV tissue was significantly decreased in failing human hearts compared to HF-free organ donors, independently of anaemia [54]. MID was associated with more pronounced coronary disease and lower beta-blocker usage compared with non-MID HF patients. Except that, MID in HF was accompanied by reduced activity of aconitase and citrate synthase as well as reduced expression of reactive oxygen species (ROS)-protective enzymes (catalase, glutathione peroxidase and superoxide dismutase 2), indicating that MID may contribute to worsening of mitochondrial dysfunction that exists in HF [54]. These findings are consistent with the recent research from Hoes et al., who demonstrated reduced energy production and contractile dysfunction in iron-deficient human cardiomyocytes [56]. Cells treated with iron chelator deferoxamine (DFO) exhibited reduced activity of iron-sulphur containing mitochondrial complexes (I, II, III) and also displayed significantly lower level of cellular ATP and reduced contractile force. Interestingly, supplementation of transferrin-bound iron reversed all functional and morphological abnormalities. Additionally, our recent research showed that intracellular iron depletion is detrimental for functioning of cardiomyocytes and skeletal myocytes and leads to increased apoptosis and reduces cell viability [57, 58].

It is also worth to notice that MID in human failing heats not only reflects reduced iron availability due to systemic iron deficiency but might also be influenced by a dysregulation of cardiomyocyte iron homeostasis, e.g. downregulation of TfR1 [18] and IRP inactivation [50]. Restoring myocardial iron content may be considered as a strategy to improve substrate utilization and myocardial energetics.

Clinical options to non-invasive detection of MID are strongly limited. Leszek et al. showed that out of all serum markers, only sTfR correlated with the depletion of myocardial iron (*r* = − 0.44, *p* = 0.03 for right ventricle and *r* = − 0.38, *p* = 0.07 for left ventricle) [55]. However, cardiac T2* magnetic resonance imaging has shown recently a potential utility for evaluating myocardial iron deficiency [59–61].

## Beneficial effects of intravenous iron supplementation in human hearts

Clinical research into a treatment of ID and anaemia in HF lead to a conclusion that the administration of erythropoiesis-stimulating agents together with intravenous iron does not yield better clinical outcomes than intravenous iron alone [62]. Further, there is no documented clinical benefit from usage of oral iron preparations [63, 64]. However, two main clinical trials (FAIR-HF and CONFIRM-HF) have proven that intravenous iron supplementation improves quality of life, exercise tolerance and reduces risk of hospitalizations for worsening of HF [65, 66]. Interestingly, the improvement in aforementioned parameters occurred in both anaemic and non-anaemic patients and was equally efficacious [67]. In smaller trials, where HF patients with ID were subjected to i.v. iron supplementation, more beneficial effects have been observed. The favourable changes have been reported in regard to both biochemical parameters such as a reduction in plasma level of NT-proBNP and the echocardiographic measures reflecting myocardial function including an increase in LVEF and attenuation in hypertrophic cardiac remodelling: reduction in LVSD (left ventricular end-systolic dimension), LVDD (left ventricular end-diastolic dimension), LVPW (left ventricular end diastolic posterior wall dimension), IVS (interventricular septal end diastolic dimension thickness), LV mass index, LV end systolic volume and in peak systolic strain rate as well as a decline in E/E0 together with an improvement in S0 and E0 [68–72].

## Conclusions

Several animal studies in which ID was induced by low-iron diet or bleeding developed numerous structural and functional cardiac abnormalities. However, because ID and IDA are inseparably related, it was difficult to establish the importance of each of these factors independently.

In this review, we summarized different approaches to induce cardiac-specific ID and discussed most important structural and functional consequences of this state. First of all, transgenic myocardial downregulation of iron import as well as iron export upregulation resulted in intracellular ID. Moreover, in a cardiac-specific TfR1 knock-out, cardiomyocytes were unable to import transferrin-bound iron into cell [31]. Furthermore, both cardiac hepcidin knock-out and loss of hepcidin responsiveness (via cardiac ferroportin knock-in) lead to uncontrolled iron export indicating that cardiac hepcidin controls cardiomyocyte iron homeostasis in an autocrine manner by regulating local ferroportin [43]. Over and above that, IRP1/2-deficient hearts exhibited intracellular ID as a result of simultaneous iron uptake reduction (transferrin receptor downregulation) and an increase in iron export (ferroportin upregulation) [50].

Regarding all transgenic mouse models discussed above, we can observe that most of heart abnormalities induced by systemic ID are also observable in the myocardial-specific ID, independently of anaemia (Table 1). All of these cardiac-specific ID models were characterized by structural aberrations, mitochondrial and metabolic dysfunctions as well as exhibited increased mortality, indicating a strong connection between MID and cardiac dysfunction. Interestingly, the severity of cardiac dysfunction differs in aforementioned animal models. This might be related to the genetic background of the mice, or more importantly to the amount of iron deficiency achieved in the hearts.

**Table 1:** Summary of animal models and clinical data investigating consequences of ID on heart structure and functioning

		Animal models					Human Heart Failure
		Systemic ID (ID diet)	Cardiomyocyte specific ID				
			*Tfr1* ^*hrt/hrt* 31^	*Hamp*^*fl/fl*^*;Myh6.Cre +* or *Slc40a1C326Y*^*fl/fl*^*Myh6.Cre +* ^43^	*Cre-Irp1/2* ^*f/f* 50^		
					Baseline	After MI	
Iron concentration in myocardium/cardiomyocytes		↓^22^	↓	↓	↓	↓	↓^18, 55, 54^
Anemia		+^22, 17, 14, 23, 13^	−	−	−	−	+/−^7^
Mortality			↑	↑	•	↑	↑^7^
Structural changes	Cardiomegaly	+^22,17, 23, 13^	+	+	•	↑	•^54^
	Cardiac hypertrophy	+ ^17, 14, 23, 13^	+	+	•	↑	•^54^
	Left ventricle dilatation	+ ^22,17, 14, 23, 13^	+	+	•	↑	•^54^
	Hypertrophied cardiomyocytes	+^14, 23, 13^	+	+	•	↑	•^54^
Systolic dysfunction		+^13^		+	+^#^	+	•^54^
Mitophagy dysfunction			+				
Apoptosis		↑^23^	↑	↑			
Mitochondria	Abnormal structure	+^23^	+	+	•		
	Number	• ^23^	↓		•		
	Mitochondrial DNA expression		↓		•		↓^54^
	Aberrant mitochondrial respiration	+^23^	+		+^#^	+	+^54^
Electron transport chain	Complex I	↓^27^	↓	↓	↓		•^54^
	Complex II	↓^27^	↓	•			•^54^
	Complex III		↓	•			•^54^
	Complex IV	↓^27^	↓	↓	•		•^54^
	Aconitase			↓			↓^54^
Metabolic derangements	Glycolysis		↑	↑			↑^54^
	Hypoxia inducible genes expression	↑ ^14^	↑				
Recovery by iron supplementation		+^13^	+	+		+	+^53^

*ID* iron deficiency, *HF* heart failure, *MID* myocardial iron deficiency, *empty cells* no data, • no differences, # acute dobutamine challenge

Interestingly, benefits of i.v. iron treatment were evident in each of the aforementioned study. Considering one of the most severe phenotype developed by cardiac transferrin receptor 1 knock-out, i.v. iron treatment prolonged the survival of mice up to 13 weeks vs. 9 days without treatment. Additionally, in cardiac hepcidin knock-out, not-binding-hepcidin ferroportin knock-in and IRP1/2 knock-out i.v. iron treatment restored myocardial ID and completely suppressed all morphological changes as well as metabolic and mitochondrial abnormalities, including even these alterations which occurred after myocardial infarction. This in turn may suggest that regardless of decrease in myocardial TfR1 expression, depleted cardiac iron store can be restored throughout i.v. iron treatment via non-transferrin-bound iron (NTBI) uptake. Moreover, continuous iron availability in the Tfr1^hrt/hrt^ knockout mice was accomplished by simultaneous knockout of hemojuvelin, which causes persistent elevation of NTBI (thereby allowing continuous iron uptake via NTBI). This strategy allowed mice to remain healthy until they were sacrificed at 12 months, showing that iron is not only essential to proper cardiac functioning, but also that it must be constantly available. Of note, there are two main potential pathways for NTBI uptake which involve divalent metal transporter 1 (DMT1) [73] and myocardial L-type Ca^2+^ channel [74] that acts as a compensatory mechanism for TfR1-mediated iron import.

The advantageous effects of iron supplementation have also been reported in clinical studies involving HF patients with ID. According to the recent ESC/HFA 2016 guidelines, all HF patients should be screened for the presence of ID, regardless of the other comorbidities [75]. In symptomatic patients with ID and HFrEF, i.v. iron supplementation should be considered to improve HF symptoms and exercise tolerance as well as quality of life [65, 66] The influence of iron repletion on improvement in morbidity, mortality and outcomes is deeply investigated in the ongoing clinical trials (FAIR-HFpEF; HEART-FID; FAIR-HF2; Affirm-AHF; MYOCARDIAL-IRON).

**Fig. 2:**
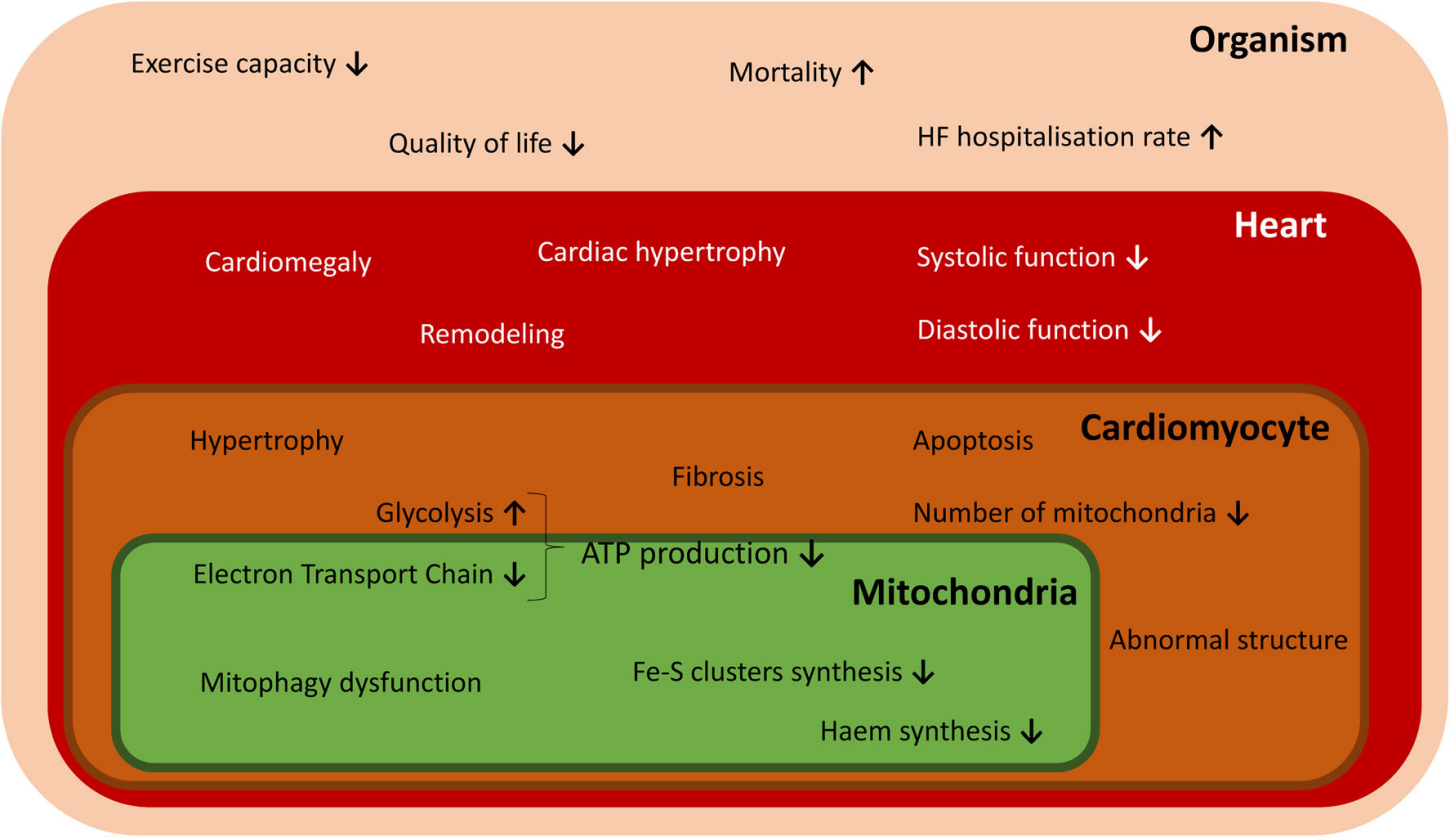
Effects of ID on structural and functional abnormalities occurring in mitochondria, cardiomyocyte, heart and the whole organism

In addition to iron supplementation, other possible therapeutic targets to restore MID in HF could be considered as alternatives. According to data from animal studies presented above, cardiac iron import may be improved by stimulation of NTBI uptake. Another therapeutic concept that is worth to be explored may involve regulation of cardiac hepcidin/ferroportin machinery which may provide blockage of iron export. Further studies investigating the exact role of local iron homeostasis in myocardium and links between ID and malfunctioning of heart are therefore warranted (Fig. 2).
